# A case series of emtricitabine-induced pure red cell aplasia

**DOI:** 10.4102/sajhivmed.v22i1.1271

**Published:** 2021-08-30

**Authors:** Nithendra Manickchund, Camille du Plessis, Melanie-Anne A. John, Thandekile C. Manzini, Bernadett I. Gosnell, Mahomed-Yunus S. Moosa

**Affiliations:** 1 Department of Infectious Diseases, Faculty of Internal Medicine, King Edward VIII Hospital, Durban, South Africa; 2 Department of Infectious Diseases, School of Medicine, University of KwaZulu-Natal, Durban, South Africa

**Keywords:** emtricitabine, pure red cell aplasia, drug induced, rare drug toxicity, adverse drug reaction, antiretroviral, anaemia

## Abstract

**Background:**

Anaemia is common in patients with retroviral disease. New or worsening anaemia after initiation of antiretroviral (ARV) treatment has a broad differential diagnosis.

**Objectives:**

We describe six patients who developed transfusion-dependent anaemia on first-line therapy (tenofovir, emtricitabine and efavirenz) and, by exclusion, implicated emtricitabine in the aetiology of the anaemia.

**Method:**

We conducted a retrospective chart review of patients seen at the Infectious Diseases specialist clinic at King Edward VIII Hospital in KwaZulu-Natal between 2014 and 2016. We focused on patients with isolated, refractory and transfusion-dependent anaemia occurring after initiation of ARVs, in whom bone marrow biopsies were consistent with pure red cell aplasia (PRCA) without an identifiable secondary cause.

**Results:**

All the patients were female, with a median (range) age and baseline CD4 cell count of 42.5 (23–61) years and 237 (83–329) cells/mm^3^, respectively. Before presenting with symptomatic anaemia, the duration on emtricitabine was 4.5 (2–8) months. At presentation, all patients had an HIV viral load of < 1000 copies/mL and a CD4 cell count of 314 (213–389) cells/mm^3^. The median time to recovery following the discontinuation of emtricitabine was 2 (1–4) months. After a median of 12 months, all patients were successfully rechallenged with emtricitabine and remained well for a follow-up period of 24 (7–36) months.

**Conclusion:**

This study provides strong circumstantial evidence that emtricitabine plays an important role in the pathogenesis of reversible PRCA. The mechanisms through which emtricitabine induces PRCA remain unclear and require further study.

## Introduction

Anaemia is common in persons living with HIV (PLWH). New onset or worsening anaemia on initiation of antiretroviral treatment (ART) has a wide differential diagnosis. Toxicity from antiretroviral drugs such as the nucleoside analogues zidovudine (AZT) and lamivudine (3TC) must be considered.

In 1998, several case reports implicated 3TC as a cause of pure red cell aplasia (PRCA).^1,2,3,4,5^ Initially, 3TC was thought to potentiate the haematological toxicity of AZT, following reports of two patients who presented with profound anaemia while on both drugs.^1^ Later, 3TC was shown to cause anaemia independently of AZT.^2^

**FIGURE 1:**
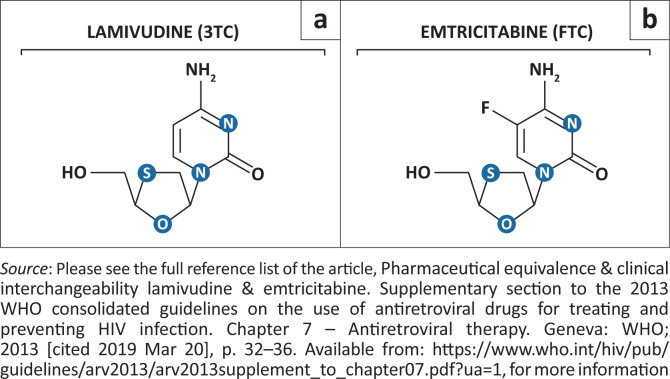
The chemical structure of (a) lamivudine and (b) emtricitabine.

There are several similarities between 3TC and emtricitabine (FTC). Both are cytosine deoxyribonucleoside analogues that target the HIV reverse transcriptase enzyme by acting as a chain terminator of viral DNA synthesis, with the only difference in their chemical structure being a fluorine atom bound to carbon 5 of the FTC pyrimidine ring (Figure 1).^6,7^ They share similar toxicities, are active against the hepatitis B virus and select the same resistance mutations. Emtricitabine, however, has a longer half-life, a higher binding affinity for the reverse transcriptase enzyme and demonstrates a greater synergy with tenofovir in inhibiting HIV replication.^6,8^ The most common mechanism of nucleoside reverse transcriptase inhibitor (NRTI) toxicity is inhibition of the human mitochondrial DNA polymerase γ enzyme, which leads to mitochondrial dysfunction. The structural differences between FTC and 3TC reduce the affinity of FTC for the γ polymerase enzyme 100-fold.^9^ This likely explains the reduced toxicity of FTC compared to 3TC.^9^

Because of the striking similarities between 3TC and FTC and the development of severe anaemia soon after ART initiation, in the absence of another cause, we suspected that FTC might be responsible for the PRCA. We present observational data from six patients with probable FTC-induced transfusion-dependent anaemia following the initiation of a fixed-dose combination of tenofovir, FTC and efavirenz.

## Method

This study was based at the Infectious Diseases specialist clinic at King Edward VIII Hospital, KwaZulu-Natal. We conducted a retrospective chart review of all patients presenting to the clinic between 2014 and 2016 with a haemoglobin (Hb) level of < 7 g/dL following the initiation of first-line ART.^10^ The department has a long-standing interest in PLWH with PRCA secondary to parvovirus B19 infection. As a result, patients with unexplained, transfusion-dependent anaemia are actively recruited for further evaluation.

Patients were included if they were aged > 18 years, infected with HIV, on an FTC-containing regimen and had isolated anaemia with a bone marrow examination consistent with PRCA, without an alternate explanation. There had to be a temporal relationship between anaemia and the use of FTC with at least 3 months’ follow-up at the clinic.

All patients underwent comprehensive investigations to determine the cause of their anaemia, including iron studies, folate and vitamin B12 levels, viral studies, a connective tissue screen and an examination of the bone marrow. The information captured included the baseline and most recent CD4 cell counts, HIV viral load, previous history of ART, comorbidities and concomitant medication use, including herbal medication. Secondary causes of red cell aplasia were excluded clinically and by directed laboratory investigations. These included pregnancy, thymoma, lymphoma, systemic lupus erythematosus, rheumatoid arthritis, juvenile chronic arthritis, multiple endocrine neoplasms and drugs such as AZT and 3TC. Infection with the following viruses was excluded: parvovirus B19, Epstein-Barr virus, human T-cell lymphotropic virus 1, and hepatitis A, B and C. Bone marrow aspirate and trephine biopsies were performed on all patients.

Patients were considered for a rechallenge with FTC once they became transfusion independent. For the rechallenge, the following criteria had to be met:

- Stable normal Hb for at least 3 months
- Fully suppressed HIV viral load for at least 3 months
- Informed consent from the patient.

### Ethical considerations

This study was approved by the University of KwaZulu-Natal Biomedical Research Ethics Committee (BREC; reference number BE586/17). This was a retrospective chart review. Because of the study design, patients’ consent was not required.

## Results

Six patients met the inclusion criteria. All were African and female, with a median (range) age of 43 (35–61) and CD4 cell count of 237 (83–329) cells/mm^3^. The median Hb at the time of ART initiation was 9 g/dL (Table 1). All patients were ART naïve before the commencement of FTC. Clinically, all patients had normal nutritional status, with a median (range) serum albumin value of 33.5 (29–38) g/L. The median time to the first transfusion after the initiation of FTC-based ART was 4.5 months. All patients had very low Hb at the initial presentation (median: 2.65 g/dL), with normal white cell and platelet counts.

**TABLE 1:** Course of pure red cell aplasia in six patients on antiretroviral therapy.

Pt	Age (years)	At ART initiation			At first presentation with anaemia							Following discontinuation of FTC		Follow-up and rechallenge			
		Hb	CD4	CD4%	Hb	CD4	CD4%	VL	Duration on FTC (months)	No. of units of PRC transfused	Hb at discontinuation of FTC post-last T/F	Further T/F	Time to recovery of Hb to >10 g/dL (months)	Time to rechallenge (months)	Duration of follow-up after rechallenge (months)	Hb at 6 months post-rechallenge	Hb at 12 months post-rechallenge
1	61	8.8	329	19.3	2.7	345	22.7	LDL	8	7	10.7	No	2	16	24	12.0	12.4
2	48	7.2	203	16.2	1.8	312	25.9	LDL	5	12	6.5	No	2	4	12	10.9	11.7
3	55	12.2	326	12.9	3.2	389	28.71	LDL	4	12	6.5	No	2	10	7	13.3	N/D
4	35	8.2	83	7.44	2.7	213	9.35	LDL	5	53	8.4	Yes	1	16	36	12.7	13.3
5	37	9.4	237	30.4	2.6	255	23.4	LDL	2	15	7.4	Yes	4	14	18	12.9	12.6
6	23	9.2	N/D	N/D	1.8	261	8.09	504	4	18	8.4	No	1	10	13	13.2	13.0

Pt, patient; N/D, not done; PRC, packed red blood cells; T/F, transfusion; VL, viral load; FTC, emtricitabine; Hb, haemoglobin; ART, antiretroviral treatment; LDL, less than the level of detection.

Note: Hb = g/dL; CD4 cell count = cells/mm^3^.

At the time of referral to investigate the anaemia, all patients had an HIV viral load of < 1000 copies/mL. Bone marrow biopsies on all patients revealed a reduction in the erythroid series, with normal to increased myeloid series. All histopathology findings were consistent with PRCA. Bone marrow culture for mycobacteria was negative for all patients.

All patients were switched from a fixed drug combination containing FTC to single agents consisting of abacavir, tenofovir and efavirenz. Following this change, all patients made a complete recovery. The median time to recovery of the Hb to 10 g/dL without blood transfusions was 2 months. Four patients did not require any further transfusions after the switch. Patient 4 required a once-off transfusion of three units of packed red blood cells within 2 weeks of the switch, and Patient 5 required a total of seven units of packed red blood cells over two admissions within the first 3 months of FTC discontinuation. Patient 6 was initially switched to abacavir (ABC) and 3TC because of a stock-out of single-agent tenofovir. Despite this, she continued to require transfusions. However, when the patient was switched to tenofovir and abacavir with permanent discontinuation of 3TC and FTC, no further blood transfusions were required, and there was a complete recovery of the Hb.

After stable recovery of the Hb and consistent good control of the HIV disease monitored over a range of 4 months to 16 months (median: 12 months), all patients were offered a rechallenge with FTC as a component of a fixed drug combination. All patients tolerated the rechallenge without consequence with up to 36 months of follow-up (median: 24 months; range: 7–36 months) (Table 1).

## Discussion

Four fundamental factors are usually considered when implicating a drug as a causal agent of an adverse event: (1) a temporal relationship between commencing a drug and the onset of the adverse reaction, (2) biological plausibility, (3) a relationship between discontinuation of the drug and the resolution of toxicity and (4) recurrence of the toxicity on rechallenge.^11^ Based on at least three of these four factors, this case series provides a compelling argument for an association between FTC, a commonly used NRTI, and the development of reversible PRCA. It is not surprising that rare toxicities only manifest in clinical practice following widespread use of the drug. This underscores the importance of ongoing pharmacovigilance after introducing a drug into clinical practice (Phase IV studies).^12^

Our findings build on a recent report of four cases from Cape Town, South Africa, implicating FTC as the cause of transfusion-dependent anaemia.^13^ Of interest, the patients in that report were all female with a median time to toxicity of 3 months, similar to our findings of 4.5 months. Interestingly, the PRCA described with the related drug 3TC has been noted in male and female patients.2^,3,5^

Patient 5 was previously published as a case report.^14^ This patient’s persistent anaemia was initially thought to be the result of parvovirus B19 infection based on a positive parvovirus B19 PCR test. Despite multiple rounds of intravenous immunoglobulin and attaining an undetectable HIV viral load with significant recovery of the CD4 cell count from 83 cells/mm^3^ to 237 cells/mm^3^, this patient remained transfusion dependent. She received 53 units of packed red blood cells over 11 months. Impressively, within 2 months of discontinuing FTC, her Hb improved to 11.5 g/dL and remained normal at the last follow-up, 2 years later. This case illustrates the importance of considering other entities when a suspected disease does not behave as expected. It is worth noting that this patient’s parvovirus B19 PCR cycle threshold, performed several times, varied between 32.97 and 37.38 cycles, suggesting a low viral load, which in retrospect was more consistent with infection than the disease.^15^ Of interest, her parvovirus B19 PCR assay remains positive more than a year after recovery at similar cycle thresholds, further supporting the unlikely role of this virus in her anaemia.

The response of Patient 6 to treatment manipulation is worth noting. The initial switch to ABC and 3TC did not substantively impact the anaemia or transfusion requirements; however, following a switch to abacavir and TDF, there was a prompt and complete recovery of the anaemia, with no further transfusions required. This suggests cross toxicity between FTC and 3TC, which is not surprising and is consistent with other reports.^13^

Several factors led us to consider rechallenging the patients with FTC after full recovery. These included the following: our observation of a prompt recovery following removal of the offending drug; FTC playing an important role in the three-drug combination treatment of HIV; FTC being a common component of most fixed drug combinations available in the state^6,16^; and frequent stock-outs of single agents, meaning that an uninterrupted supply of alternates to FTC would be a challenge.

Failure of the recurrence of the toxicity following rechallenge makes for interesting speculation on the pathogenesis. We posit that the marrow requires a double hit, the first being bone marrow that has been made vulnerable by pre-existing anaemia or uncontrolled HIV with immune dysregulation, and the second hit being the drug, which, together with the appropriate host genetic background, leads to toxicity. Uneventful rechallenge following adequate recovery of the bone marrow suggests that the presumed genetic predisposition is necessary but not sufficient for the toxicity to manifest and that uncontrolled viral replication or immune dysregulation play an important role.

The limitations of this report include its retrospective design and small case numbers. The inferences about causality are strengthened by the temporal relationships with onset and recovery of the toxicity; however, it is important to note that this remains an uncontrolled observation.

## Conclusion

This study provides strong circumstantial evidence that FTC plays an important role in the pathogenesis of reversible PRCA in a subset of patients with HIV commencing ART. This entity should be considered when common causes of refractory anaemia are excluded. The mechanisms through which FTC induces PRCA remain unclear and require further study.
